# How Bridging Approaches Further Relationships, Governance, and Ecosystem Services Research and Practice

**DOI:** 10.3390/su17094177

**Published:** 2025-05-06

**Authors:** Kathleen C. Williams, Leah M. Sharpe, Sebastian Paczuski, Keahna Margeson, Matthew C. Harwell

**Affiliations:** 1 Great Lakes Toxicology and Ecology Division, USEPA Office of Research and Development, Duluth, MN 55804, USA; 2 Gulf Ecosystem Measurement and Modeling Division, USEPA Office of Research and Development, Gulf Breeze, FL 32561, USA; 3 Oak Ridge Associated Universities, Duluth, MN 55804, USA; 4 School for Resource and Environmental Studies, Dalhousie University, Halifax, NS B3H 4R2, Canada; 5 School of Planning, Dalhousie University, Halifax, NS B3H 4R2, Canada; 6 Pacific Ecological Systems Division, USEPA Office of Research and Development, Newport, OR 97365, USA

**Keywords:** boundary work, ecosystem services, environmental governance, geographic context, stakeholders, translational ecology

## Abstract

Understanding environmental governance empowers researchers and practitioners alike to work towards solutions that improve both environmental and human well-being outcomes. Collaborative, iterative approaches to governance use bridging approaches such as translational ecology, boundary work, and ecosystem services. The US Environmental Protection Agency’s Office of Research and Development worked with a variety of collaborators to implement six multi-year coordinated case study research projects. The research projects were designed to support agency collaborators spanning different geographies, ecosystems, and environmental management decision contexts, and to demonstrate that different tools, approaches, and ecosystem service foci can enhance coastal and other water resource sustainability. To better understand the iterative and collaborative nature of the cases and collaborations, researchers conducted an analysis of the comparative case studies based on Williams’ (2018) and Ostrom’s (1994, 2009) frameworks. The team identified (1) who participated in the processes; (2) what the programs and goals were; (3) where programs worked and their resources; and (4) the resulting outcomes. We demonstrate that stakeholder participation and outcomes look different within different projects, and we conclude that relationships, focus on place, and common goals produce the most impactful results.

## 1. Introduction

Understanding environmental governance empowers researchers and practitioners alike to work towards solutions that improve both environmental and human well-being outcomes. Within sustainable environmental governance systems, responsibility for natural resource management is shared between an array of federal and sub-federal governments and non-state actors, including nonprofit organizations and businesses as well as the public [1-3]. The literature on environmental governance increasingly highlights the importance of collective action for resource management [4]. Frameworks such as Elinor Ostrom’s Institutional Analysis and Development (IAD) and social-ecological systems (SES) were developed to characterize and advance our understanding of specific elements of collective management, including the biophysical, social, and institutional dimensions [5,6]. While IAD, SES, and other systems management frameworks provide a crucial base for understanding interrelationships and social-ecological dynamics, frameworks are only useful if they help explain what we observe [7].

There are numerous collaborative approaches for environmental governance [8], many of which include iterative processes [9], practice-based knowledge [10], and bridging concepts to build trust and create space for learning [11]. Another concept that has been an important integrating concept is ecosystem services, which refers to the benefits humans receive from nature [3,12,13]. In their review of environmental governance theories, Partelow et al. (2020) point out that while there is a positive trend towards using integrated or bridging theories, such as SES [6], there is rarely critical reflection on framework application [7]. The resulting uncertainty about applied and theoretical effectiveness leaves a gap in our understanding about the strengths, weaknesses, and generalizability of collaborative management frameworks.

To address this gap, we undertook a comparative case study of six place-based research projects conducted by researchers at the Environmental Protection Agency’s Office of Research and Development (EPA ORD). The EPA ORD developed six multi-year coordinated research projects to advance ecosystem goods and services (EGS) science in distinct geographic and program settings with different outcomes. The projects are unique because they took place at long-term EPA ORD research sites, five of which are located in coastal zones on the Pacific and Atlantic Oceans, Gulf Coast, and Great Lakes. (According to the US Coastal Zone Management Act, the US Great Lakes are considered coastal zones.) By comparing the cases, we gathered insights about (1) how decision contexts shift and are defined in practice; (2) the EPA ORD’s contributions to multiple decision dimensions (i.e., regulator, funder, researcher); (3) stakeholder identification, definition, and roles; and (4) the desired outcomes and how they are defined by different participants. We contend that these long-term research projects present a model for developing relationships that demonstrate how to advance ecosystem service science while adapting to different types of governance.

## 2. Background

### 2.1. Science and Decision Making

Lemos and Agrawal (2006) argue that environmental governance and interventions aimed at changes in environmental and scientific knowledge, decision making, and behaviors are one and the same [3] (p. 298). Although scientific knowledge is essential for informed decision making in environmental protection and governance, there is a perceived divide between science, policy, and resource management decisions [14]. While scientific information informs evidence-based policy or practice, questions have been raised regarding what constitutes “useful knowledge” [15] and knowledge sharing. Owens, Petts, and Bulkeley (2006) and Whitmer et al. (2010) point to disconnects between science communication models and the messiness of policymaking and preexisting priorities to help explain the science–policy divide [16,17]. They argue that current “prescriptions for closing [the] gap” rely too heavily on a simple “linear model of knowledge transfer” to improve communication between researchers and policy makers [16] (p. 633). Closing the gap will require rethinking communication approaches, including systematically acknowledging the messy realities of agency priorities. Translational ecology is one bridging approach that seeks to close the gap by bringing together ecologists, decision makers, and others to address on-the-ground management issues [18]. Translational ecology is committed to “real world outcomes” [18] and can provide opportunities for scientists to provide technical support to decision makers.

Inspired by translational medicine, which seeks to improve the application speed of bench research to patient care, translational ecology aims to traverse the science–use gap by addressing mismatches between scientific research supply and practitioner demand. Translational ecology expands the scope of engagement beyond use of scientific research to specifically address decisions, as co-defined by practitioners, that can be informed by research [18]. There are many reasons information may not be used by practitioners, including that information relies on preconceived notions or is at the wrong scale or resolution to provide answers to existing problems [16,19]. Translational ecology prioritizes increasing information use through multi-way learning that builds trust and develops mutual understandings of context, interests, and cultures [19,20]. When using translational ecology, scientists and decision makers engage in relationship building, as decision makers learn how science can improve their work and scientists learn the nuances of policy and constraints on decision makers [19,21].

There are many approaches for implementing translational ecology, including boundary work (another bridging approach), or the objects or concepts that facilitate working across different types of expertise or experience [22]. Another commonly used definition of boundary work is “those acts and structures that create, maintain, and break down boundaries” [23]. Individuals and organizations engaging in boundary work must “mediate between scientists and decision-makers on the one hand, and between these actors at different scales on the other” [24]. The goal of boundary work is the production of usable information by scientists for decision makers. According to Wall et al. (2017) and others, usable information must be relevant, credible, and legitimate [12,21,22]. Scientific research can produce relevant, credible, and legitimate information in many ways, including highly collaborative processes (i.e., coproduction) and those that are less intensive (i.e., consultation) [21,25,26]. Specific methods to create usable knowledge include consultation, joint fact finding, or structured decision making, and iterative dialogue [19,27]. Place or physical features can be effective boundary objects [28,29]. It is important to acknowledge that it is difficult to measure the success of translational efforts. Ideally, one could point to a specific decision or outcome and directly trace it back to a corresponding translational ecology approach; however, this involves long time frames and external influences [18]. Over time, translational approaches may lay the framework for even deeper engagements and relationship building to create a shared understanding of the environmental stressors and resources through traditional ecological knowledge and social sciences [30].

### 2.2. Community Values and Decisions

**Figure 1.**
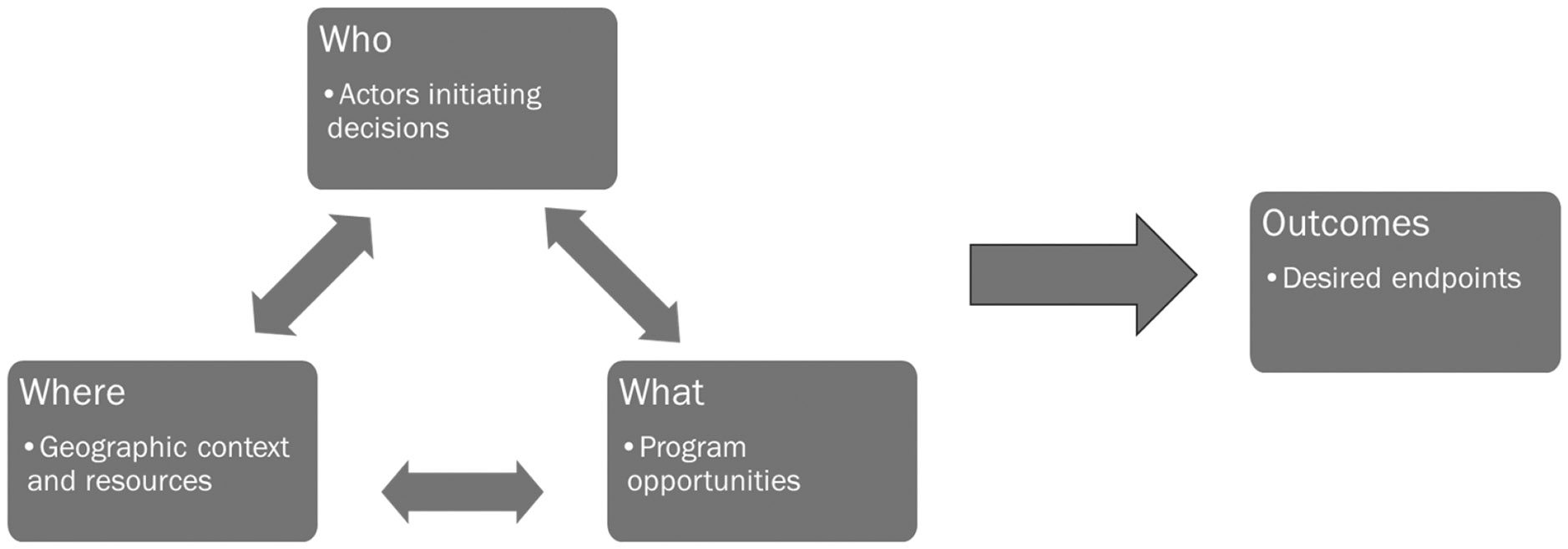
Framework for understanding how key elements create different conditions in the decision processes (adapted from Williams et al. (2018)) [31].

EPA ORD researchers conducted research designed to assess how community values and contextual factors such as a range of decision options, geography, and relationships between community and agencies were related to the use of scientific research in decisions. Williams et al. (2018) used an exploratory case study methodology to identify and characterize these relationships [31]. The researchers found that overlapping decisions might be made in geographic proximity to each other, but the environmental projects, decision makers, and desired ecosystem services and benefits were shaped by the decision context (e.g., aquatic clean-up, brownfields remediation, comprehensive planning) [32] (Supplemental Figure S1). An analysis of these overlapping decision contexts revealed that program representatives responsible for decision making (*who*) determined what decisions they would make, *where* the work would happen, which funding or administrative mechanisms (*what*) they could use, and their desired *outcomes* (Figure 1).

Understanding contextual elements is important because different EGSs provides different information to different stakeholders at different decision-relevant points. For example, Williams et al. (2018) concluded that improving EGSs was the end goal for the aquatic remediation and restoration decisions made through environmental programs [31]. EGSs provided opportunities for communities to revitalize and improve human well-being through community development [32]. Outcomes, success, and stakeholder participation can look markedly different depending on the type of project or environmental action. Thus, it was important to develop methods and concepts that can be adapted to meet the needs of decision makers, such as boundary work and translational ecology [18,22], to better meet the information needs of practitioners. Moreover, it was important to characterize how governance (e.g., program requirements and comprehensive or natural resource management plans), stakeholder input, and desired EGSs could be used to guide the actions of researchers and decision makers.

### 2.3. EPA ORD Research

#### 2.3.1. Ecosystem Services Case Studies

**Figure 2.**
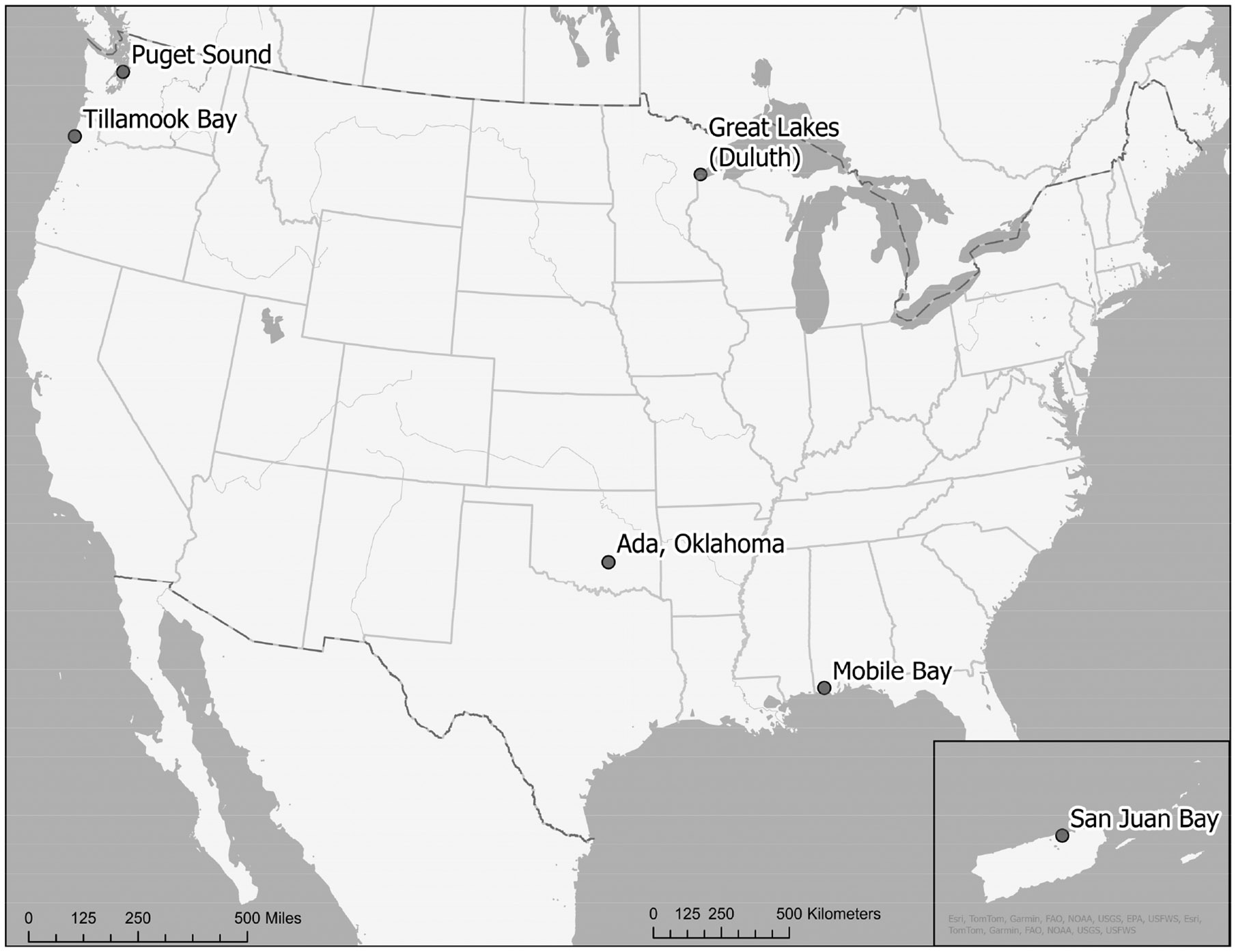
Map of six EPA ORD research case study locations.

This study analyzed six completed EPA ORD ecosystem services case studies. Five of these case studies lie in coastal zones, where a National Estuary Program (NEP) organization was a major partner [33]. The study sites included a habitat restoration site in a freshwater estuary in the US Great Lakes (Duluth, Minnesota); watershed management to improve salmonid habitat in watersheds upstream from the coast (Puget Sound, Washington); estuary management and health impacts (San Juan Bay); land–water connections and habitat in an estuary (Tillamook Bay, Oregon); aquifer management (Ada, Oklahoma); and watershed management in Mobile Bay, Alabama (Figure 2). These cases were chosen because they are place-based studies, ecosystem services are a key concept for collaboration, and tools are used according to the needs of the information users.

The case studies started with similar objectives of incorporating EGSs into community decision making and used a structured decision-making approach. Both objectives were applied to conduct research in substantially different contexts, making them particularly representative for this analysis. Results from the case studies have been summarized and reported previously [27,34]. The synthesis reports compile *practical strategies* for final ecosystem goods and services (FEGS) applications in community-based decision support and demonstrate the elements of structured decision making [35,36]. The six case studies provide valuable data on EGS research undertaken in individual cases, an inventory of the structured decision-making approaches used, and characterization of a range of EGS benefits (Table 1).

#### 2.3.2. Case Study Site Descriptions

##### 1. Great Lakes, Minnesota

The Great Lakes case study research included a Health Impact Assessment (HIA) of a habitat restoration project in the St. Louis River Areas of Concern (AOC) in the St. Louis River Estuary in Duluth, Minnesota. The St. Louis River is the largest US tributary to Lake Superior, the world’s largest freshwater lake by surface area.

The EPA ORD researchers engaged in the restoration effort by providing resources to conduct an HIA, a six-step process that uses data from different sources to demonstrate how a policy (e.g., a habitat restoration) will change health determinants to affect health outcomes [37]. The HIA worked with stakeholders (i.e., state, local, and Tribal resource managers), including the community, to identify the health determinants that would be impacted by the project. Seven health determinants or pathways were assessed: water quality, noise pollution, light pollution, transportation and traffic, recreation and aesthetics, social and cultural, and crime and safety. As part of the HIA process, recommendations were made for a project design that would reduce the negative construction impacts on the neighborhood, enhance recreational and social opportunities, and reduce crime. The Minnesota Department of Natural Resources (MNDNR) adopted many of the recommendations.

##### 2. Puget Sound, Washington

The Puget Sound case study research identified watershed management practices in forests that improve habitat for threatened salmonid populations, provide clean drinking water, support local forest sector jobs, and provide cultural benefits for local communities and Tribes. Puget Sound is an inlet of the Pacific Ocean located along the northwestern coast of Washington state. Extending about 100 miles north-to-south, Puget Sound is the second-largest estuary in the United States, containing 19 contributing watershed sub-basins with 18 major rivers draining into Puget Sound.

EPA ORD researchers working with watershed managers in the Puget Sound region used the EPA-developed Visualizing Ecosystem Land Management Assessments (VELMA) tool with stakeholders to determine beneficial watershed and forest management practices [38]. Stakeholders that collaborated on applications of VELMA included Nisqually Community Forest stakeholders (community members, the Nisqually Tribe, and Washington Department of Natural Resources), the Snoqualmie Tribe, the Oregon Department of Fish and Wildlife, and the US Forest Service. The collaborative modeling work resulted in the adoption of specific forest best management practices in Nisqually Community Forest, floodplain salmon habitat restoration recommendations considered by the Snoqualmie Tribe, and the Oregon Department of Fish and Wildlife’s use of VELMA as a conservation plan guiding tool.

##### 3. San Juan Bay, Puerto Rico

The San Juan Bay, Puerto Rico, is a bay on the island of Puerto Rico that faces the Atlantic Ocean. Case study research was focused on developing information to communicate the potential impacts of estuary management decisions on social, economic, and ecological benefits to the community living within the San Juan watershed using a Human Well-Being Index framework [39]. San Juan Bay is a major estuary that runs through the city of San Juan, located on the main island’s northeastern coast.

EPA ORD researchers worked closely with the San Juan Bay Estuary Program (SJBEP) to support their management plan, which aims to restore and conserve the estuary’s water quality while supporting economic and recreational activities [40]. Stakeholders for the case study work included the SJBEP, the Martin Peña communities, and an ENLACE community group. Stakeholders were engaged to identify knowledge gaps where research was needed, helped obtain data for models through citizen science and partnership efforts, and were updated and debriefed on research results. The case study demonstrated transferability of the Human Well-Being Index to culturally and geographically distinct populations. Researchers also found they demonstrated the value of an ecosystem service assessment in the later stages of a structured decision-making process to inform and build public support for work underway.

##### 4. Tillamook Bay, Oregon

The Tillamook Bay case study research focused on examining information gaps relevant to shellfish harvest closure decisions related to the identification of suitable habitats for bay clams and characterizing environmental drivers of elevated fecal bacteria levels based on land use and hydrology. Tillamook Bay, Oregon (34 km^2^; average depth 2 m; surrounding watersheds 1546 km^2^), is the second largest estuary in the state, containing tidal channels and extensive sand and mud flats.

EPA ORD researchers worked extensively with the Tillamook Estuaries Partnership (TEP), an organization within the EPA’s National Estuary Program that serves as a coordinator for addressing major issues facing coastal communities. The TEP consists of a large suite of partners and collaborators, including the Oregon Departments of Fish and Wildlife (ODFW), Agriculture (ODA), and Environmental Quality (DEQ) and their collective focus on sustainable production of shellfisheries. The ORD researchers collaborated with the TEP, ODFW, ODA, and ODEQ to design and lead projects to determine locations of suitable habitats for bay clams and examine environmental drivers of elevated concentrations of fecal bacteria. These efforts were designed to address knowledge gaps identified in the TEP’s Comprehensive Conservation and Management Plans [41]. The outcomes of this case study included demonstrating new methods for modeling and GIS-based mapping of suitable habitats for five shellfish species and describing the hydrologic and land-use contributions to seasonal and spatial differences in fecal bacteria. The results were peer-reviewed and published to make available to the TEP and the Oregon State agencies involved with shellfishery decision making.

##### 5. Mobile Bay, Northern Gulf Coast

The Mobile Bay case study research, on the Gulf Coast, examined a proposed restoration project in the D’Olive sub-watershed coordinated by the Mobile Bay National Estuary Program (MBNEP) designed to restore natural flow, reduce downstream turbidity, and improve fish and wildlife habitats. The Mobile Bay watershed (113,084 km^2^, spanning four states) includes the major metropolitan area of Mobile, Alabama, which supports a major port and shipyard and commercial and recreational fisheries.

Rather than directly influencing restoration planning for the D’Olive sub-watershed, EPA ORD researchers asked to participate in the MBNEP-led restoration project and used their processes to test ecosystem service tools and approaches (e.g., VELMA and associated tools to examine ecosystem services and water storage changes over time) for the estuarine and shoreline components of the project. EPA ORD researchers used models to examine potential changes in ecosystem services anticipated from the restoration project (e.g., clean water, water storage, recreation, and access to greenspace) to support restoration assessments and examine utility for use in future restoration planning. The case study’s primary stakeholder was the Mobile Bay National Estuary Program (MBNEP), an organization within the EPA’s National Estuary Program that serves as a coordinator for addressing major issues facing coastal communities [33]. Working with partners, the MBNEP brings together citizens, government agencies, businesses, environmental organizations, and academic institutions to develop and provide information to protect the integrity of Mobile Bay. The case study outcomes were used to guide the development of a “stressor matrix tool” to be used in the future by the MBNEP and partners to prioritize identified “stressor-service links” for future restoration and conservation planning efforts.

##### 6. Ada, Oklahoma

The Ada, Oklahoma study aimed to develop and evaluate water management options that would best provide for the long-term sustainability and resiliency of the city of Ada and surrounding communities that draw their source water from the Arbuckle Simpson Aquifer using the EPA-developed Decision Analysis for a Sustainable Environment, Economy and Society (DASEES) tool [42]. Ada is located in south central Oklahoma. The region is mainly rural, with Pontotoc County forming part of the Chickasaw Nation’s territory and the City of Ada serving as both the Chickasaw Nation headquarters and the county seat.

EPA ORD researchers conducted two sets of workshops implementing the DASEES Structured Decision Making tool. The first two-day workshop in June 2018 partnered with the City of Ada, Ada Water Resources Board, East Central University, and Oak Hills Golf and Country Club to inform development and resource management planning to address the challenges facing the City of Ada and the community water users that rely on and receive their water from the Arbuckle-Simpson aquifer through the City of Ada water treatment and distribution system. The second two-day workshop was held in November 2019 with an expanded scope to address regional drought contingency planning with communities and others that rely on and draw their water from the Arbuckle-Simpson aquifer. The second workshop considered information gained from the first workshop with the City of Ada and added in the perspectives of the expanded number of stakeholders at the workshop. This expanded regional focused workshop included representatives from the City of Ada, Chickasaw Nation, Arbuckle Karst Research Group, Oklahoma Water Resources Board, Oklahoma Department of Environmental Quality, Oklahoma Department of Wildlife Conservation, National Park Service, Oka’ Institute, Ada Jobs Foundation, Blue River Foundation of Oklahoma, Arbuckle Master Conservatory District, Nature Conservatory, East Central University, and South Central Climate Adaption Science Center. Researcher and partner work on modeling, analysis, and decision making for planning implementation currently continues for both of these groups.

## 3. Methodology

We used a comparative case study approach [43]. Case studies are often used when phenomena are the object of study (e.g., ecosystem services and tools) and cannot be separated from their contexts. Case studies are also useful when answering “how” and “why” questions [44]. We undertook extensive document analysis with NVivo 12Pro [45] to determine how contextual factors (e.g., who, what, where) were related to EGS outcomes. These factors are often embedded in everyday decisions and thus require a “thick description” that is an “interdisciplinary analytical approach which is pluralistic in its consideration of different values that inform decision making” [46] (p. 1106).

In addition to document analysis, we held EPA staff debrief sessions, which filled in gaps and provided insights about EPA ORD scientists’ experiences working on each project. Analyzed documents included EPA public reports, workshop or meeting notes, project files, and research plans (see Tables S1 and S2). Many of the project documents were co-authored with non-EPA or non-EPA ORD collaborators. The document types varied across the case studies because of differences in research team operations, project initiation, and project execution. This is not a limitation, but rather an aspect of the data that added richness and nuance to the analysis. The number of documents available for review varied at each site, and a sample of seven to ten documents was selected for comparability (see Tables S2 and S3) and analyzed.

We used content analysis to identify and characterize critical case elements that influenced outcomes, with particular attention to the role of stakeholders [47]. We deductively coded using a codebook (see Table S3) derived from Williams et al. (2018) to uncover the individual case elements and stakeholder involvement as they occurred [31]. This type of analysis is valuable because it revealed how scientists and decision makers navigated these complex processes. Based on our results, we discuss how these case elements are related to case outcomes.

Our results are presented by theme in the Who-What-Where-Outcomes framework (i.e., similar to Ostrom’s IAD and SES frameworks [5,6]) descriptively. This approach was chosen because, as Enquist et al. (2017) noted, it is difficult to draw straight lines from a particular action to a result in collaborations [18]. This approach allows for a thicker description and to identify and characterize the nuance embedded in the case study research because the case studies displayed different arrangements of researchers, partners and collaborators, decisions, and approaches to support.

## 4. Results

### 4.1. Who and What Role

#### 4.1.1. Stakeholders

All six case studies identified and engaged stakeholders [48] or individuals or groups who possess an interest, obligation, right, or concern in the decisions or outcomes of decisions relating to the projects. There was no one definition of stakeholders across the studies. The Great Lakes study considered stakeholders to be organizations or agencies with an interest in the outcome of a project within a specific geographic area. This was similar to how the Mobile Bay study defined stakeholders, namely those that were generally located or working within the D’Olive watershed. The Tillamook Bay study defined stakeholders more programmatically as including the Tillamook Estuaries Partnership (an NEP) and their partners (a long list identified for other purposes), state natural resource and environmental quality management agencies (e.g., ODFW, ODA), and recreational and commercial shellfish harvesters. Similarly, the Puget Sound study (categories that included communities, Tribes, state and federal agencies, and private organizations) and the Ada, Oklahoma, project (communities, municipalities, Tribal nations, decision makers, and a project steering committee with agency representation) focused on a systematic assemblage of stakeholders. The San Juan Bay study identified stakeholders more broadly, focusing on including those living, working, or operating within the San Juan Bay estuary watershed as stakeholders.

The six case studies had different approaches to inviting stakeholders to participate. The Great Lakes; San Juan Bay; Puget Sound; Ada, Oklahoma; and Tillamook Bay studies all mentioned explicitly inviting stakeholders to meetings and/or workshops for different purposes. The Great Lakes study invited stakeholders to share feedback at various project phases and on project results. In the San Juan Bay, stakeholders were invited to identify knowledge gaps and engage with a citizen science effort. In Puget Sound, stakeholders were invited to participate in modeling workshops and trainings. In Tillamook, stakeholders were invited to help design the Comprehensive Conservation Management Plan. Interestingly, the Mobile Bay study had no references to stakeholders being invited to participate.

Primary methods for longer-term stakeholder engagement include a project leadership team and advisory committee in the Great Lakes case study; an informal working group in the Mobile Bay NEP; and a project steering committee in the Ada, Oklahoma, case study. When stakeholders were not engaged, it mattered. In the Mobile Bay study, researchers noted that a lack of stakeholder engagement early in the process led to missing data later:

“Stated consequences were tightly aligned with the NEP CCMP [Comprehensive Conservation and Management Plan] goals and excluded measures of human benefit such as recreation, health, or economic improvements. Valuation attempted to consider increases in property values and public safety but lacked sufficient data. These things were not prioritized and might have been if a stronger stakeholder engagement exercise had preceded the work”.

#### 4.1.2. Collaborators

Collaborators were defined in the study as people, groups, organizations, and agencies who work together on a project or aspects of a project. They can overlap with stakeholders, but they are not necessarily the same groups. Collaborators are important because they indicate power or work sharing. Collaborators were identified across all of the case studies. The Tillamook study referenced collaborators the most, while the Great Lakes study had more types of collaborators, including a community group, the EPA ORD, state government, and Tribal partners. The Great Lakes case study demonstrated a significant level of partnership and collaborator interaction. The San Juan Bay and Mobile Bay studies also referred to collaborators, while the Ada, Oklahoma, study had the fewest references to collaborators.

All six case studies had several groups of collaborators in common, including the EPA ORD, a state government agency, and a university partner. Community groups, environmental non-governmental organizations, and the EPA Regional Offices were identified in all but the Ada, Oklahoma, study. A local governmental agency was identified in all of the case studies, except in the San Juan Bay study.

Community groups were important collaborators. The Tillamook Bay study identified community groups as the most frequently cited collaborator; this finding reflected the work of the Tillamook Estuaries Partnership and their engagement with many community user or interest groups. The Puget Sound and Great Lakes studies also had a high number of community groups identified; the Great Lakes study identified community groups as regular, integral partners for their work. The San Juan Bay case study identified community groups as important partners. The Mobile Bay study mentioned community groups while the Ada, Oklahoma, study did not engage community groups. Interestingly, the Mobile Bay study’s stakeholder matrix referred to the need for “strong public stakeholder infrastructure”, suggesting that those roles are filled by environmental non-governmental organizations and local government.

The four National Estuary Program studies (Tillamook Bay, Mobile Bay, Puget Sound, and San Juan Bay) had few or no references to federal agencies outside of the EPA, but engaged a range offices within the EPA, including the EPA ORD, Regional Offices, and Program Offices.

#### 4.1.3. Communications

Communication approaches (i.e., how groups were engaged) among the case studies included episodic and continuous communication approaches, as well as one-way and two-way efforts. The Great Lakes and Tillamook studies explicitly identified one-off (one time only) communications. For example, the Great Lakes study referred to signage demarcating culturally significant places that could be installed at the project site. The Tillamook Bay study referred to research manuscripts on research findings (e.g., EPA products) as a one-off communication example.

All of the case studies, except Mobile Bay, demonstrated ongoing communication with stakeholders and collaborators. In general, the Great Lakes; San Juan Bay; Puget Sound; Ada, Oklahoma; and Tillamook Bay’s ongoing communications efforts included engaging stakeholders and residents from local communities in the research process through a variety of methods, including ongoing meetings, the presentation of findings, requests for input, collaborative manuscript writing, committees, and conference calls. Often, these ongoing communications were specific to the topic of the case study. In the Puget Sound study, this included working with and teaching people how to use the VELMA model. In the Ada, Oklahoma, study, communication included ongoing efforts to re-engage stakeholders to participate in parts of the HIA. In the Tillamook Bay study, communication included sharing information with Tillamook Estuaries Partnership members; ongoing education and outreach; opportunities for open discussions; an accessible, interactive, and updated website; and appearances and engagement in the community.

One-way communication was prevalent in all of the case studies, particularly that which took place between the EPA ORD and other project partners (senders) and community members, stakeholders, partners, and decision makers (receivers). In the Great Lakes, San Juan Bay, and Tillamook Bay studies, these communications took the form of briefings, journal articles, reports, and manuscripts. In Ada, Oklahoma, the EPA ORD and project partners were trying to reengage municipal decision makers to participate in water planning. Other examples of one-way communication in the Great Lakes study included meeting notifications and flyers, invitations to meetings, and recommendations to project partners from the EPA. In the Tillamook Bay study, one-way communication included sharing information about the Tillamook Estuaries Partnership and their bylaws, vision, and goals, which included the transfer of information to partners, public input at meetings, and interactive website elements. Interestingly, the Great Lakes study was the only one with a community-member-to-EPA one-way communication pathway, where recommendations were shared with the EPA.

Two-way communication involving information transfer from the sender to the receiver and vice versa was present in all of the case studies except Mobile Bay. The Great Lakes study demonstrated a structured two-way communication process between decision makers and stakeholders and community members, providing intentional opportunities for feedback, deliberate engagement, and back-and-forth interactions. The Puerto Rico; Puget Sound; and Ada, Oklahoma, studies demonstrated less structured methods for establishing ongoing stakeholder involvement, engagement, and opportunities for feedback, starting early in the process and continuing throughout. The Tillamook Bay study referenced communication with the Tillamook Estuaries Partnership which included learning from partners, open discussions, and consultation. Co-authorship manuscript reviews from partners and key stakeholders were another example of two-way communication evident in the Tillamook Bay study.

### 4.2. What Programs or Decisions

#### Decision Context

The EPA ORD case studies were experiments that supported external partners to address key management needs through the development and application of tools and use of ecosystem services. It was through supporting partners that the EPA had the opportunity to (co-)develop, test, and operationalize approaches and tools related to ecosystem services. As a result, the opportunities and experiences of the research teams varied. Some research topics were the result of long-term collaborations, while others were newly formed partnerships. Understanding this background is key for analyzing the decision context of the coordinated case studies.

The goals for the research efforts fall along three different spectra (1) the number of research questions or management problems being addressed; (2) the level of collaboration (and relatedly, EPA ORD input); and (3) the level of integration between management efforts and research design.

In the Great Lakes study, for example, EPA ORD researchers, state agencies, and community members co-defined the problem. This was possible because EPA ORD researchers were embedded in several connected management efforts, including (1) the Great Lakes Water Quality Agreement through the AOC program; (2) removing beneficial use impairments through the implementation of a Remedial Action Plan; (3) specific management action project plans; and (4) an HIA for informing the decisions of the Minnesota Department of Natural Resources and City of Duluth, Minnesota. This integration was the result of long-term collaboration with stakeholders, and it meant that the potential contributions of EPA ORD research and the needs of the decision makers were both considered when defining the case study work.

In contrast, in the Mobile Bay study, EPA ORD researchers contributed their expertise and experience to support priorities identified by local and regional partners who were developing an ORD. Researchers saw how their work could benefit decision makers who were developing the Watershed Management Plan for the D’Olive Watershed and the National Estuary Program’s Comprehensive Conservation and Management Plan. Both groups of partners and the research team had wanted to prioritize resilience and climate change in their plans as important elements that could be addressed. The case study research focused on this single goal, one that had been identified by the National Estuary Program, but the case study work was conducted independently. In this case, EPA ORD researchers used this knowledge of the management needs to provide decision support and conduct research for the managers’ consideration and to better demonstrate a suite of approaches that could be beneficial in their planning process. The EPA ORD researchers’ priorities and expertise contributed to the specific approaches selected and how they were applied. This case study is a product of long-term research in ecosystem services and decision making and was aiming to begin the development of a new external collaboration by combining that research with completed and ongoing work carried out by the NEP in their Comprehensive Conservation and Management Plan and Watershed Management Plan.

The San Juan Bay study was involved in many of the decisions that the NEP was trying to address, including an HIA [49,50], NEP management actions, and other planned decisions. Stressors the NEP was trying to address included carbon and nitrogen cycling and harmful algal blooms, fungal conditions in homes, and ecosystem goods and services. The EPA ORD case study team in Puerto Rico had been working in the San Juan Bay NEP long term on different projects, including a Human Well-Being Index [39] and identifying ecosystem services. As a result, the team was embedded and able to connect with the community that had specific information needs and build on an earlier HIA. The case study researchers addressed multiple research questions, some co-developed with the NEP. While research goals were set in response to NEP needs and influenced by community concerns, the specific research questions were defined in large part by the EPA ORD.

In the Puget Sound study, decision makers co-defined the problem in helping managers make decisions and inform stakeholders. This work arose from a long-term research project investigating how to use models to examine impacts, drivers, and the trade-offs of management actions, and to help with decision making and technology transfer. This case study emphasized methods for estimating benefits and examining tradeoffs that support decision making as well as stakeholder communication. The EPA ORD’s desired research context and the decision context were nearly identical. However, the EPA ORD focused more on understanding issues around data use by others and the transferability of approaches to other settings in addition to the immediate needs of the local agency.

The Ada, Oklahoma, study focused solely on water management for the City of Ada, Oklahoma, a priority defined by local decision makers. The EPA ORD researchers’ role was to provide decision support with the Decision Analysis for a Sustainable Environment, Economy, and Society (DASEES) [51] tool. This case study represented a new collaboration for this long-term tool development research project. The lead local management agency organized consultation with stakeholders, including Tribes, agencies, and community members, whose input was used to articulate the bounds of the decision context.

While stakeholders were engaged in the Tillamook Bay work, there were no specific managerial actions or plans the case study was designed to support. EPA ORD researchers connected multiple studies and experiments to higher level problems and supporting the NEP (e.g., by identifying nutrient sources to help improve water quality in the face of climate change). The Tillamook Bay case study demonstrates how long-term climate and land-use-change research can support development of the NEP Comprehensive Conservation and Management Plan’s goals through ongoing relationship building.

Through our analysis, we see management partners and EPA ORD researchers defining a vast array of decision contexts. The Mobile Bay study was focused on the NEP’s Comprehensive Conservation and Management Plan and Watershed Management Plan, while the Ada, Oklahoma, study was focused on issues of water management as defined by the City of Ada, Oklahoma. In contrast, the Great Lakes study referenced different types of decisions, processes, and legal instruments, while the San Juan Bay study referenced several previous, planned, or ongoing decisions and actions. The Puget Sound study was interesting in that there was an overall focus on improving decisions and informing stakeholders, but a wide range of specific research questions were identified under that umbrella.

### 4.3. Where: Place and Resources

#### 4.3.1. Geographic Context

Geographic context was important in all of the case study sites. This finding makes sense because ecosystem services cannot be separated from their biophysical context and location. Site description was identified in our analysis as the most important factor in the most complex case studies—Great Lakes; Ada, Oklahoma; and Tillamook Bay. For example, in the Great Lakes study, the geographic context was defined by overlapping geographies, including the Great Lakes Areas of Concern Program, which encompasses the Great Lakes Region; the St. Louis River Area of Concern in Minnesota and Wisconsin; and the restoration site geography. Moreover, the project specifically focused on how the ecosystem services provided by the St. Louis River would change based on habitat restoration. Thus, the site and biophysical environment were critical to understanding the potential decisions made. The Puget Sound Restoration Program was described using a variety of geographic terms, including Puget Sound, the Salish Sea, forests, and watersheds. The VELMA model is an important model to connect the land use in the upland forests to the health of the streams and river that flow into Puget Sound. Finally, in the Tillamook Bay study, Tillamook Bay, as an environmental resource, along with the watersheds that feed into the estuary, were mentioned both specifically and in general.

The Ada, Oklahoma, case study demonstrated the importance of its geographic context through an extensive discussion of the Arbuckle-Simpson aquifer. Ada, Oklahoma, is in the Great Plains region of the United States, a region that frequently experiences water scarcity, leading communities to explore cooperative water management approaches. The San Juan Bay and Mobile Bay case studies focused on specific sites: the Martin Peña Canal and Forest Lake, respectively. In addition to general discussion about geographic context, three specific themes emerged—boundaries (e.g., the extent of forests, watersheds, and estuaries), ecology (e.g., species and land-water connections), and landscape features (e.g., specific places and objects of study).

#### 4.3.2. Ecosystem Services

How EGSs were discussed varied greatly across the case studies. Ecosystem services were especially important in the Great Lakes study because ecosystem services and social determinants of health were important elements in the HIA. On the other hand, EGSs were mentioned only a handful of times in the Mobile Bay study. EGSs had different meanings for each study. For example, the Great Lake case study references to EGSs ranged from beneficial uses of the environment to specific ecosystem services such as recreational use. In the Tillamook Bay study, EGSs were specific to the coastal ecosystem, including bivalves and seagrass. In the Puget Sound study, EGSs were flood protection, cultural resources, and salmon habitats. In the Puerto Rico study, the EGSs were both material objects (e.g., timber) and ecosystem functions that could benefit humans (e.g., carbon sequestration, climate regulation, reduction in disease vectors). The Ada, Oklahoma, study focused solely on water resources and available drinking water as EGSs.

The question of who determined the ecosystem focus varies by case study. In the Great Lakes study, ORD scientists, the HIA team, and community members determined which EGSs would be considered. In the Mobile Bay study, there were very few references to how EGSs were defined, though information gathered through community surveys and EPA ORD scientists’ expertise contributed. In the Puerto Rico study, there were many groups who defined EGSs, including stakeholders, partners, state and federal agencies, and local governments. In the Ada, Oklahoma, study, the EGSs under investigation were determined by the communities participating in the study. Finally, in the Tillamook Bay study, the NEP, communities, and EPA ORD researchers defined the EGSs.

### 4.4. Outcomes

The six case studies focused on developing or applying approaches for the use of EGS concepts to state-agency- or community-level decision-making. All of the case studies were developed with the goal of transferring approaches, frameworks, and tools to other communities and users with different environmental decision contexts. As such, the outcomes of this work were assessed for transferability, utility, scalability, and communication. We examined three types of outcomes in our analysis: (1) tangible products, (2) outcome applications, and (3) research results/findings. The spectra that were uncovered in the decision context analysis (the number of research questions, level of collaboration, and level of integration) were useful for exploring these results as well.

Through our analysis, we identified four types of tangible products (Table 2). Some of the products are program-reporting work for the NEPs and others are required EPA ORD reports. As scientists, the EPA ORD publishes journal articles. Finally, there are communications specifically requested by the stakeholders.

We found that the case studies addressing more research questions and that were the result of longer-term collaborations (Great Lakes, San Juan Bay) tended to produce a higher quantity and variety of products than case studies that were focused on a single issue or a newer collaboration (Ada, Oklahoma; Mobile Bay).

We also explored how the case study outcomes were applied to support environmental decision making, and those applications can be grouped into four categories (Table 3).

Outcomes were more likely to be acted upon, rather than purely informative, when case studies were built on longer collaborations, when research questions were coproduced between the EPA ORD and partners, and/or when research and planning or decision-making processes were integrated. In the long term, collaboration and extensive cooperation in the Great Lakes study created opportunities to apply the frameworks and model approaches developed over time to help discuss the ecosystem service and decision context to inform current and future environmental decision making by interpreting the values held by stakeholders, comparing across boundaries of experience, and facilitating communication. As a result, stakeholders considered recommendations from analyses of project alternatives and integrated them into project design and implementation. Additionally, beyond the tactical recommendations, overall guidelines for the stakeholder project changed because of the effort.

In the Mobile Bay and Ada, Oklahoma, studies, on the other hand, where the case studies were the first step in developing new collaborations, results had limited application. In the Mobile Bay study, results were shared with the partners and stakeholders and focused primarily on providing information for examining priorities (e.g., where to carry out restoration within the watershed) for new projects. The project informed ecosystem assessments after the fact, a key first step for demonstrating the value of new approaches to partners. Currently, the Mobile Bay NEP is working on integrating EGSs into future monitoring, and researchers are focusing on quantifying possible benefits using models for future consideration in other sub-watersheds. In the Ada, Oklahoma, study, results informed decision-making efforts and supported the development of monitoring and adaptive management plans, with an eye towards transferability to other decision contexts. The EPA ORD is also investing in improving the ease with which results are shared and communicated by refining how results are displayed (e.g., generating plots, charts, and graphs of the data) with attention to the making the results understandable by a lay audience (i.e., one without special scientific or technical knowledge).

Similarly, the Tillamook Bay study’s collaboration was less developed, and the outcomes have been more informative than actionable. The case study research results were shared with the NEP, but the process for state agencies to alter their monitoring methods (e.g., shellfish stock assessment, fecal bacteria) or decision criteria (e.g., harvest closures) takes time. For example, the ODA is considering using a case study model estimating likely exceedance concentrations for fecal bacteria based on environmental drivers (wind, rain, tide, and location within Tillamook Bay).

In the more developed collaborations in the San Juan Bay and Puget Sound studies, the results were targeted at both managers and stakeholders. In the San Juan Bay study, the focus was on linking restoration to improvements in human health and communicating those potential benefits to local communities. In the Puget Sound study, the focus was on transferring knowledge and tools for partners and stakeholders to use in future decision making and in regulatory and policy contexts. Examples of these applications include the following:

- Informing advocacy for the dredging of the Martin Peña canal (the San Juan Bay study).
- Examining tradeoffs between environmental clean-up solutions (reducing urban trash, dredging the canal, improving water quality in the estuary) versus traditional pesticide spraying (the San Juan Bay study).
- Applying a new San Juan Bay Human Well-Being Index for local- and national-level decision and policy making (the San Juan Bay study).
- Connecting salmon recovery modeling to forest management decisions with the Nisqually Tribe (the Puget Sound study).
- Integrating terrestrial–marine ecosystem service modeling frameworks for informing Puget Sound recovery planning (the Puget Sound study).
- Informing oil spill trajectories and contaminant circulation for US–Canada policy discussions (the Puget Sound study).

In general, the research results from the six case studies focused on three thematic areas (1) specific results from models and frameworks, (2) the applicability of models and EGS concepts to inform decisions, and (3) findings related to the technology transfer of models and tools to stakeholders. Consistent with the patterns for products and applications, we found that case study documentation of research results increased as collaborations became more developed. Broadly, the Great Lakes study documentation contained the most content related to research results; the Mobile Bay and Ada, Oklahoma, studies contained the least; and the San Juan Bay, Puget Sound, and Tillamook Bay studies fell in between.

## 5. Discussion

Our analysis of these case studies identified some interesting patterns across the critical case study elements that were illuminating and would be worth consideration in research and practice (Table 4). Although all six studies were place-based, place seemed to play a more important role in some of the case studies than others. Similarly, EGSs, which may be a function of place, played a larger role in some case studies than others. Geographic context and EGSs were both the most important elements in the Great Lakes case study, a site where there were a large range of stakeholders who had many different attachments to the environmental resources. In the Puget Sound and Tillamook Bay studies, the geography and site description were also central to the research. These conditions may have helped make the research more responsive to the needs of the practitioners, where their work is often tied to a specific geography. This is important because EGSs and place can be integrative concepts or boundary concepts. There were also relationships between researchers and practitioners where the research was based on the EGSs of the specific decision context. Thus, the geographical context (i.e., place) and EGSs functioned as boundary concepts (i.e., a common focus) to create a closer relationship between the researchers and practitioners based on a shared understanding of the problem to solve.

**Figure 3.**
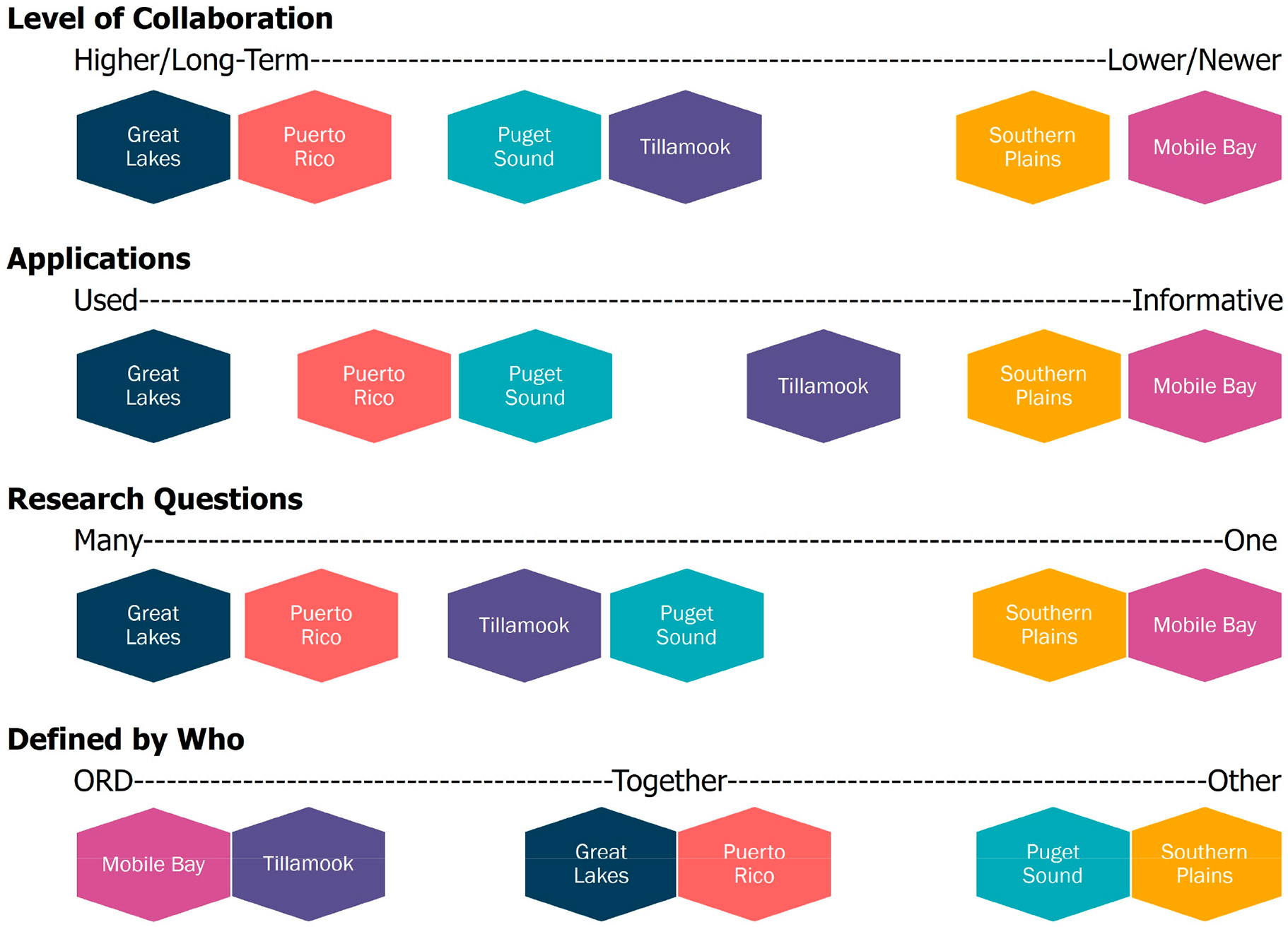
Depiction of case study elements and relationships as scaled to depict diversity of experiences in EPA ORD case studies.

Quite prominently, the length of the ORD–partner collaboration is associated with several aspects of the case studies’ decision contexts and outcomes. Across these six case studies, the efforts in which the collaboration between the researchers and partners was extensive and long-standing (Great Lakes, San Juan) were also efforts in which (1) the research questions were co-developed by both researchers and external partners, (2) multiple research questions were being addressed, and (3) the research outcomes were used in some way by the partners (Figure 3). These connections have a logic to them. As the collaborative relationship between researchers and partners deepens, the researchers gain a better understanding of the partners’ needs, and the partners gain a better understanding of the researchers’ capabilities. Additionally, as a collaboration continues, trust between the parties and a shared set of expectations can develop. This improved understanding and increased trust allows each party to become involved in the other ‘s planning processes and supports the co-development of research questions that address management needs as well as agency priorities. A co-development process with knowledgeable participants allows for the identification of additional research questions that would otherwise be unexplored due to lack of knowledge about manager needs or research capabilities. Increased trust also supports the exploration of multiple research questions, as the parties have greater expectations that engaging in those efforts will be a good use of their time. Co-developed research questions that are tied into planning processes and are responsive to specific needs also increase the likelihood that the research results will be both useful and used.

The goal of many researchers engaging in place-based work is to see their study results applied in decision making. The results of this analysis suggest that there is an association between the strength of collaboration and this desired result. This analysis also suggests that place-based work in which the collaboration is newer or in which the collaboration is being established might benefit from considering different definitions of project success (i.e., establishing partnerships, where specific ecosystem outcomes may come later). In our analysis, there were two case studies in which researchers were working to establish a collaboration for the first time (Figure 3; Mobile Bay and Ada, Oklahoma). In these case studies, the work focused on a single research question developed unilaterally (by researchers in Mobile Bay and partners in Ada, Oklahoma) and the research results were used for informational purposes rather than used in decision making. A comparison of those elements, however, is incomplete without consideration of the collaboration context. Given the importance of strong collaboration in achieving applied results, our analysis suggests that measures of success related to collaboration development would be informative for “early-collaboration” case studies. For these case studies, focusing on a single, clearly defined question and on communicating results so that partners see the potential of future work could be a key step in deepening the collaboration.

The closer research is to a partner ‘s problem, the more the research will be salient and credible (Figure 3) [12,22]. Closeness cannot be forced, and case studies with less established relationships should consider where they are on the spectrum of collaboration as they design their project and set their research goals, as well as setting goals related to the collaboration itself. Our results suggest that the effectiveness of place-based work is tied to the strength of collaborative relationships. Partner collaboration is a long-term investment and collaboration planning should be considered alongside research planning with the goal of moving research as close to a partner’s problem as possible.

There are some important implications for this research. For example, we saw the principles of translational science at work in the case studies [18,21]. In several case studies, we saw how researchers working closely with collaborators, including state agencies, communities, and Tribes, resulted in products that met the needs of the users and were applied in practice. For example, the Great Lakes research team communicated with decision makers, the MNDNR, and the City of Duluth to understand their decision context before beginning the HIA. Throughout the research, the team engaged the MNDNR and City of Duluth through the iterative process; thus, their information needs were addressed throughout the HIA process. Similarly, the Puget Sound research team employed a coproduction method [26] where the Tribes and communities and state agencies were part of the modeling development process, thus integrating partner needs throughout the development process. Additionally, the Puget Sound team conducted ongoing trainings for potential users of the VELMA and Salish Sea models. What these examples demonstrate is that translational ecology, more than a nice idea, is imperative to create products that meet the informational needs of potential users and to create research that can be applied in a decision context.

Boundary work was an important component of this research. It is essential that we recognize that decision or policy realms and research might be distinct, and that they both have identities that should be retained. Thus, it is important to engage in boundary work where efforts can be made to ensure that both the decision makers and researchers are conducting their work and completing their goals. Meaningful collaboration where research and decision integrity were both respected can be seen, especially in the Great Lakes; San Juan Bay; Ada, Oklahoma; and Tillamook Bay case studies. In those cases, the boundary work looked like researchers participating in decision processes and supporting the decisions that were being made. Because the researchers were part of the decision process, they were able to tailor the research to ensure that it was impactful and spoke to the needs of the decision makers. The important lesson learned here is that it is important for researchers to understand the decision context, to have relationships with the decision makers, and to consider the information needs of the decision makers when crafting their research questions. At the same time, researchers also conducted hypothesis-driven research alongside the decision-relevant research; thus, the roles, responsibilities, and identities of the researchers and the decision makers were maintained.

The most important lesson learned through this analysis is that researchers must carry out the groundwork to build relationships and understanding as well as recognize the information needs and program requirements of their collaborators. But clearly, Figure 3 illustrates that there is no single “right” answer or method for working with stakeholders and collaborators. The opportunities to work with stakeholders and collaborators may be a product of time, opportunities, and meaningful engagement. In some case studies that are just beginning this long-term process (i.e., Mobile Bay), the team may not have the relationships or understanding needed to fully engage in translational methodologies. However, as the team continues to work with the Mobile Bay NEP, relationships will deepen, understanding will be built, and research questions can be developed that more closely reflect the informational needs of the NEP. The translational research that was implemented in the Great Lakes and in the San Juan Bay was the product of nearly a decade of working with decision makers and stakeholders. What this contrast demonstrates is that this approach will result in progress over time, that research questions will reflect their new knowledge of stakeholder and collaborator needs, and that research will then be more easily applied in those decision contexts.

## 6. Conclusions

This study has been an opportunity to look deeper into the patterns of the EPA ORD’s coordinated case studies. We wanted to conduct this study because we recognized that the different case studies had different outcomes and relationships with other parts of the EPA, state agencies, or other environmental programs, and we wanted to know what contributed to the successes of some of the case studies. We used an adaptation of Ostrom’s IAD and SES frameworks developed from social science research as an analysis method to help identify and characterize different relationships and how they contributed to both ecosystem service research and outcomes. More specifically, we wanted to know *who* participated in the research? *Who* were the participants and how were they contributing to or applying the research? *What* programs were the EPA ORD research case studies working with? *Where* was the research conducted and how important was place to the problem to be solved? How were stakeholders and collaborators engaging in the research? What were the desired *outcomes* for the ORD and the programs?

Our findings point us to two main conclusions. The first conclusion is that relationships matter in research and research application. While this seems intuitive, it was important to empirically examine this. In the case studies where the researchers from the ORD had close relationships (i.e., trust, ongoing engagement) with the agencies or programs that would apply the research, the research was more often accepted and applied to the decisions on the table for the agency. In other cases, where the relationships are not as well established and are still in the formation stage, it might take several years before research findings that have meaning for the stakeholders can be developed. With continued investments over time, we expect that researchers and agencies will develop a mutual understanding of how ecosystem services and tools could help solve the problems they are trying to solve. The second conclusion is that creating relationships around common interests through boundary work and translational science in the place where practitioners work on the EGSs they manage [27,28] is foundational to ensuring that both scientific research and practice-based knowledge is applied to solving mutual problems that may have multiple desired outcomes. Our research demonstrates Enquist et al. (2017)’s finding that there are many approaches to implementing translational ecology [18]. Thus, we conclude that it is imperative to develop closer relationships to build a common understanding of program needs and research opportunities to ensure more effective collaboration and research and program outcomes. This requires making a decision to dedicate an investment in time, two-way communication, coordination, and efforts to co-develop a shared vision of the project and its desired outcomes.

## Supplementary Material

Supplement1

**Supplementary Materials:** The following supporting information can be downloaded at: https://www.mdpi.com/article/10.3390/su17094177/s1, Figure S1: Diagram of how different EPA programs have different policy mechanisms to affect different types of projects; Table S1: List of documents analyzed in the study; Table S2: Number of documents and pages coded in the study; and Table S3: Codebook used in the study.

## Figures and Tables

**Table 1.** Summary of important case elements.

Site Name	Location and Program Details	Research Case Study Goals	Partners	Case Study Research Methodology or Approach
Great Lakes	Duluth, MN St. Louis River Estuary Great Lakes Water Quality Agreement Area of Concern	Identify health determinants impacted by AOC restoration project	MN Dept Natural Resources MN Pollution Control Agency City of Duluth	Modeling and ecosystem service mapping Stakeholder engagement and participatory mapping exercise to gather input from with community members; Tribal, professional, and scientific experts Tool: Health Impact Assessment
Puget Sound	Puget Sound Inlet Northwest coast of WA National Estuary Program	Identify watershed management practices in forests for improving stream conditions for salmon habitats, drinking water, local jobs, and cultural benefits	Nisqually Tribe WA Dept Natural Resources Snoqualmie Tribe OR Dept Fish and Wildlife US Forest Service	Tool: VELMA modeling; stakeholder collaboration and application
San Juan Bay	San Juan, Puerto Rico San Juan Bay Estuary National Estuary Program	Communicate impacts of estuary management decisions on social, economic, and ecological benefits to San Juan watershed population	San Juan Bay Estuary Program (SJBEP) Martin Peña communities ENLACE Community Group US Army Corps of Engineers	To support San Juan Bay CCMP Tool: Human Well-Being Index
Mobile Bay	Mobile, AL Mobile Bay Estuary D’Olive Watershed National Estuary Program	Test ecosystem tools and approaches in context of MBNEP-led restoration projects	Mobile Bay National Estuary Program (MBNEP)	To support Mobile Bay CCMP Tool: VELMA (and associated tools); FEGS Scoping Tool
Tillamook Bay	Tillamook Bay Estuary, OR Northwestern coast of OR National Estuary Program	Examine shellfish harvest closure, decision making, and information gaps, and characterize environmental drivers of fecal bacteria	Tillamook Estuaries Partnership (TEP) OR Dept of Fish and Wildlife OR Dept Agriculture OR Dept Environmental Quality	Shellfish habitat suitability modeling study Fecal bacteria environmental drivers study Incorporated data from stocking assessments and monitoring Tool: VELMA
City of Ada	Arbuckle-Simpson aquifer Regional water use	Develop and evaluate water resource management options for the City of Ada	City of Ada, OK Chickasaw Nation Oklahoma Water Resources Board	Tool: DASEES

**Table 2.** Types of tangible products produced through comparative case study research.

Products	Where Applied
Reports aligned with existing reporting mechanisms	The three NEPs: Tillamook Bay, Mobile Bay, San Juan Bay
Other EPA ORD reports	Great Lakes, San Juan Bay, Mobile Bay
Peer-reviewed journal articles	San Juan Bay, Great Lakes
Stakeholder-focused communication deliverables	Great Lakes; Tillamook Bay; San Juan Bay; Ada, Oklahoma

**Table 3.** Case study outcome application by decision makers.

Learning or Knowledge Outcomes	Where Applied
Influencing philosophies, goals, and strategies	Great Lakes
Contributing to decision making	Great Lakes, San Juan Bay, Puget Sound
Informing future regulatory or policy context	Great lakes, Puget Sound, Tillamook Bay
Informing future science efforts	Tillamook and Mobile Bays

**Table 4.** Major findings from case study analysis and comparison.

Major Findings
Geographic context or place is an important consideration because EGSs may be a function of place.
Longer ORD–partner collaborations resulted in research findings being applied more often.
When research is more closely aligned with partner needs, it is more salient, credible, and reliable—or usable.

## Data Availability

The data for this study are the list of documents, numbers of pages coded, and our codebook. All of the data can be found in the Supplemental Materials.

## Abbreviations

The following abbreviations are used in this manuscript:

AOC: Area of Concern in the Great Lakes
DASEES: Decision Analysis for a Sustainable Environment, Economy, and Society
EGS: Ecosystem goods and services
EPA ORD: US Environmental Protection Agency Office of Research and Development
FEGS: Final ecosystem goods and services
HIA: Health Impact Assessment
IAD: Institutional Analysis and Development
MBNEP: Mobile Bay National Estuary Program
MNDNR: Minnesota Department of Natural Resources
NEP: National Estuary Program
ODA: Oregon Department of Agriculture
ODEQ: Oregon Department of Environmental Quality
ODFW: Oregon Department of Fish and Wildlife
SES: Socio-ecological system
SJBEP: San Juan Bay Estuary Program
TEP: Tillamook Estuaries Partnership
VELMA: Visualizing Ecosystem Land Management Assessments
