# Supplementary material for: How Bridging Approaches Further Relationships, Governance, and Ecosystem Services Research and Practice
Source: Sustainability. Author manuscript; Available in PMC 2026 May 6. (PMC12774332; doi:10.3390/su17094177)
Supplement: Supplement1 [file NIHMS2122369-supplement-Supplement1.pdf]

Supplementary Material

How Bridging Approaches Further Relationships, Governance, and Ecosystem Services Research and Practice

Kathleen C. Williams [1,*], Leah M. Sharpe [2], Sebastian Paczuski [3], Keahna Margeson [3,4,5] and Matthew C. Harwell [6]

[1] Great Lakes Toxicology and Ecology Division, USEPA Office of Research and Development, Duluth, MN 55804, USA

[2] Gulf Ecosystem Measurement and Modeling Division, USEPA Office of Research and Development, Gulf Breeze, FL 32561, USA

[3] Oak Ridge Associated Universities, Duluth, MN 55804, USA

[4] School for Resource and Environmental Studies, Dalhousie University, Halifax, NS B3H 4R2, Canada

[5]School of Planning, Dalhousie University, Halifax, NS B3H 4R2, Canada

[6]Pacific Ecological Systems Division, USEPA Office of Research and Development, Newport, OR 97365, USA

*** Correspondence:**
Corresponding Author
williams.kathleen@epa.gov

1 Supplementary Figures and Tables

1.1 Supplementary Figure

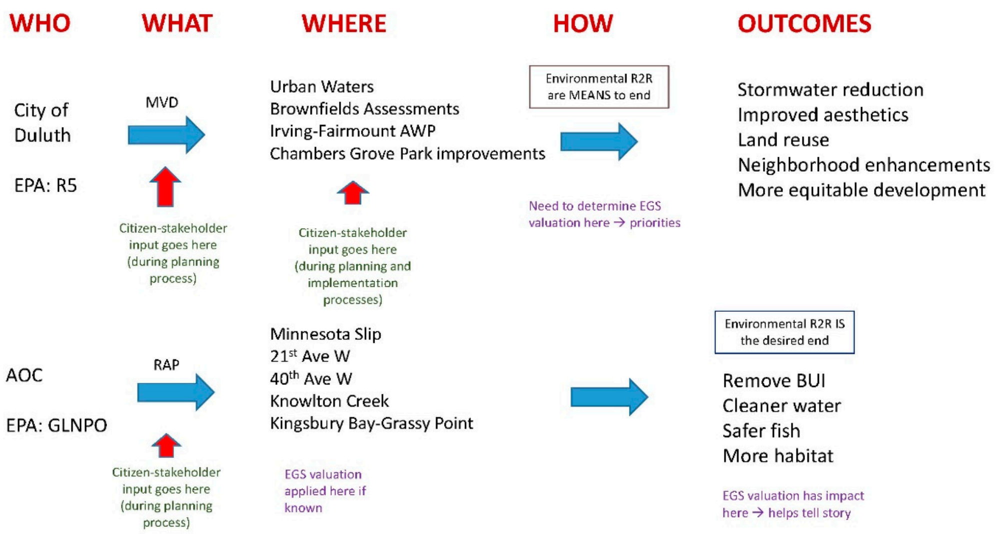

Supplementary Figure S1. Diagram of how different EPA programs have different policy mechanisms to affect different types of projects. Ecosystem services and community or stakeholder input may influence the process at different times resulting in different desired outcomes. EPA: R5 is EPA Region 5. EPA: GLNPO is the EPA Great Lakes National Program Office. MVD was a program called Making a Visible Difference, where EPA actions were directed to move the needle on intractable problems in communities. RAP is a Remedial Action Plan, or a systematic plan to tackle the overlapping environmental problems of legacy aquatic contamination and habitat loss in formerly industrial areas. The significance of this diagram is that there are different EPA programs that operate in adjacent or overlapping spaces with different objectives and mechanisms for action. Similarly, there are different opportunities to consider ecosystem goods and services, and different opportunities to consult with the public. As a result, even when programs appear to have similar goals – the ultimate desired outcomes may differ.

1.2 Supplemental Tables

Supplementary Table S1. List of documents analyzed in the study.

CCS Site	Document Title	Document Type	Agency/Organization	Author(s)
Puerto Rico	Understanding Effects of Urbanization on Ecology and Ecosystem Services of a Subtropical Estuary	Presentation slides	EPA, NHEERL, Gulf Ecology Division	Rose Martin, Cathleen Wigand, Alana Hanson, Autumn Oczkowski, Stephen Balogh
Puerto Rico	An EPA Pilot Study Characterizing Fungal Populations at Homes with Flooding Events at the Martin Pena Channel Community	Project management plan	EPA, NHEERL, Gulf Ecology Division	
Puerto Rico	Predicting Effects of Climate and Landuse Change on Human Well-Being via Changes in Ecosystem Services	Presentation slides	U.S. Environmental Protection Agency, Gulf Ecology Division	Susan Yee, Elizabeth Paulukonis, Jessica Orlando, Cody Simmons, Linda Harwell, Marc Russell, Richard Fulford, and Lisa M. Smith
Puerto Rico	An EPA Pilot Study Characterizing Fungal and Bacterial Populations at Homes after Flooding Events at the Martin Peña Channel Community – Puerto Rico	EPA public report	ORD, NRMRL, APPCD, IEMB	Doris Betancourt, Timothy Dean, Stephen Vesper, Evelyn Huertas
Puerto Rico	Dynamic Modeling of Ecosystem Services and Human Well-being Endpoints in the San Juan Bay Estuary Area	Presentation slides	EPA, NHEERL, Atlantic Ecology Division	Stephen Balogh
Puerto Rico	Technical Guidance for Constructing a Human Well-being Index (HWBI): A Puerto Rico Example	Model or tool	EPA, NHEERL, Gulf Ecology Division	Jessica L. Orlando, Susan H. Yee, Linda C. Harwell, and Lisa M. Smith

Puerto Rico	Ecosystem Services Coordinated Case Study: San Juan, Puerto Rico	Program background	EPA, NHEERL, Gulf Ecology Division	
Puerto Rico	Quality Assurance Project Plan: San Juan Coordinated Case Study	QAPP	EPA, NHEERL, Gulf Ecology Division	Susan Yee
Puerto Rico	Shrinking Cities Examined from a Shrinking Scale: Neighborhood Homogeneity and Impacts on Material and Energy Consumption, Ecosystem Services and Environmental Impact	Presentation slides	EPA, NHEERL, Atlantic Ecology Division	Stephen Balogh, Justin Bousquin, Elvia Melendez-Ackerman, Cecilio Ortiz, Tischa Munoz-Erickson, Ariel Lugo, Susan Yee, Gustavo Garcia-Lopez, Marla Perez Lugo
Mobile Bay	D'Olive Watershed: Path Toward Restoration	Land use or resource management plan	D'Olive Watershed Working Group	
Mobile Bay	2016 Quality Assurance Project Plan: Gulf of Mexico Coordinated Case Study	QAPP	EPA, Gulf Ecology Division	Richard Fulford, Rebeca de Jesus Crespo, John Rogers, Marc Russell, Kate Murphy, Elizabeth Paulukonis, Susan Yee, Leah Sharpe
Mobile Bay	Ecosystem Services Research: Mobile Bay Watershed	Presentation slides	EPA, Gulf Ecology Division	Richard Fulford, Leah Sharpe
Mobile Bay	Mobile Bay National Estuary Program Program Implementation Committee Meeting	Meeting notes and minutes	Mobile Bay National Estuary Program	
Mobile Bay	Mobile Bay National Estuary Program Science Advisory Committee Meeting	Meeting notes and minutes	Mobile Bay National Estuary Program	

Mobile Bay	The Values: What do Citizens Value about Living on the Coast?	Website	Mobile Bay National Estuary Program	
Tillamook Bay	Tillamook Estuaries Partnership, State of the Bays 2015: Health Report	Program background	Tillamook Estuaries Partnership	
Tillamook Bay	Tillamook Case Study	Presentation slides	EPA, Costal Ecology Branch	Ted DeWitt, Cheryl Brown, Jim Kaldy, Darryl Marois, Chris Mochon Collura, Steve Pacella, Jody Stecher, Nate Lewis, Chanda Littles, Amy Simmer-Faust, York Johnson, Alex Manderson
Tillamook Bay	Tillamook Estuaries Partnership Bylaws	Program background	Tillamook Estuaries Partnership	Chris Knutsen
Tillamook Bay	Ecosystem Response and Recovery in PNW Estuaries: Tillamook Case Study	QAPP	EPA, Western Ecology Division	
Tillamook Bay	Habitat Utilization and the Effects of Water Quality on Harvested Shellfish	QAPP	EPA, Western Ecology Division	Ted DeWitt
Puget Sound	Summary of VELMA Modeling Projects and Partners in Region 10	Model or tool	EPA, Western Ecology Division	Bob McKane
Puget Sound	A Science-Governance Partnership for Integrating Ecosystem Services into Puget Sound Restoration Planning	Presentation slides	EPA, Western Ecology Division	Bob McKane, Brad Barnhart, Paul Pettus, Jonathan Halama, Allen Brookes, Kevin Dijang, Tarand Khangoankar, Isaac Kaplan, Chris Harvey, Hem Nalini Morzaria Luna, Michael Schmidt, Emily Howe, Philip Levin, Tessa Francis, Joel Baker, Stephen Stanley, Colin Hume

Puget Sound	Vision Statement for a Puget Sound Basin Coupled Environmental and Human Systems Modeling Framework	Manuscript draft	EPA, Western Ecology Division	Bob McKane, Brad Barnhart, Paul Pettus, Jonathan Halama, Allen Brookes, Kevin Dijang, Tarand Khangoankar, Isaac Kaplan, Chris Harvey, Hem Nalini Morzaria Luna, Michael Schmidt, Emily Howe, Philip Levin, Tessa Francis, Joel Baker, Stephen Stanley, Colin Hume
Puget Sound	Puget Sound Partnership: 2018-2022 Action Agenda Proposed Near Term Action	Project management plan	Puget Sound Partnership, EPA, Western Ecology Division	
Puget Sound	Urban Watershed Modeling in Seattle, Washington using VELMA- a Spatially Explicit Echohydrological Watershed Model	Abstract	EPA, Western Ecology Division	Brad Barnhart, Bob McKane, Paul Pettus, Jonathan Halama, Paul Mayer, Allen Brookes, Kevin Djang
Puget Sound	How Visualizing Ecosystem Land Management Assessments (VELMA) Modeling Quantifies Co-Benefits and Tradeoffs in Community Forest Management	Presentation slides	EPA, Western Ecology Division	Bob McKane, Jonathan Halama, Paul Pettus, Bradley Barnhart, Allen Brookes, Kevin Djang, Greg Blair, Justin Hall, Joe Kane, Paula Swedeen, Laurie Benson
Puget Sound	Tolt Modeling Update Workshop	Presentation slides	EPA, Western Ecology Division	Bob McKane, Jonathon Halama, Chris Vonderasek, Nadia Seeteram
Puget Sound	VELMA Modeling QAPP WED	QAPP	EPA, Western Ecology Division	Bob McKane
Ada	Ada's Water Supply- The Path Forward	Land use or resource	Ada Water Resources Board	Dick Scalf, Guy W. Sewell

		management plan		
Ada	Challenges of Arbuckle-Simpson Study	Presentation slides		
Ada	Developing Work with the City of Ada	Program background	EPA ORD	
Ada	Decision Analysis for a Sustainable Environment, Economy & Society	QAPP	EPA NRMRL	Bryan Dyson, Randy Parker, Jim Voit, Tim Canfield, Marilyn TenBrink
Ada	Project Scope	Project management plan	Ada Water Resources Board	Guy W. Sewell
Ada	2.61 Coordinated Case Study- Southern Plains Case Study Questions	Meeting notes and minutes	EPA	Katie Williams, Leah Sharpe, Matt Harwell
Ada	Ecosystem Services Coordinated Case Study: Southern Plains	EPA public report	EPA	Tim Canfield
Great Lakes	St. Louis River AOC, Grassy Point R2R Plan	Program background	EPA MED	
Great Lakes	How the community value of ecosystem goods and services empowers communities to impact the outcomes of remediation, restoration, and revitalization projects	EPA internal report	EPA MED	Kathleen Williams, Joel Hoffman, David Bolgrien, Ted Angradi, Jessica Carlson, Rosita Clarke, Adam Fulton, Heidi Timm-Bijold, Molly MacGregor, Anett Trebitz, and Salaam Witherspoon
Great Lakes	Kingsbury Bay - Grassy Point HIA Stakeholder Kick-Off Meeting Notes	Meeting notes and minutes	EPA MED	

Great Lakes	KBGP Habitat Restoration: HIA Community Kick-off Meeting Notes	Meeting notes and minutes	EPA MED	
Great Lakes	Community Invite - Stakeholders	Workshop materials	EPA MED	
Great Lakes	KBGP Findings and Recommendations Posters	EPA public report	EPA MED	
Great Lakes	Mud Lake Ecosystem Services Technical Brief	EPA public report	EPA MED	
Great Lakes	Mud Lake Future Alternatives Community Values and Health Impact Assessment	EPA public report	EPA MED	
Great Lakes	Mud Lake HIA QAPP	QAPP	EPA MED	Kathleen Williams, Joel Hoffman, Ted Angradi

Supplementary Table S2. Number of documents and pages coded in the study.

**Site**	**Documents submitted / selected**	**Total pages coded**
Puerto Rico	47 / 9	324
Mobile Bay	7 / 6	63
Tillamook Bay	5 / 5	242
Puget Sound	10 / 8	175
Ada	20 / 7	97
Great Lakes	171 / 9	154

Supplementary Table S3. Codebook used in the study.

Codes and Subcodes	Definitions
**Purpose**	**Organizational and bureaucratic reasons for which the CCS research projects were created or continued by EPA; including ties to larger programs, past research, and requests from Program or Office Partners**
StRAP (Strategic Research Action Plan)	References associating the CCS research to specific StRAP programs, tasks and sub-tasks.
Program_Regional support	References to requests from Regional or Program Office partners, or to the CCS research as a byproduct of relationships with Regional or Program Office partners.
Past research	References to CCS research being built on previous ORD research. May build on earlier work on a similar topic at the same site, but not necessarily a continuation of previous projects.
Longterm project	References associating the CCS research with a long-term ORD research project.
Personal research	References associating the CCS research with scientist(s) personal interest in a topic.
Non-EPA origin	References to documents and plans that did not originate within EPA, but are part/all of why projects were created or continued by EPA
**Geographic and Biophysical Context**	**Geographic conditions associated with CCS research projects and project sites**
Geographic scope_extent	References to the general geographic scope within which the CCS research extends.

Site description	References to descriptions of specific CCS research project sites.
Boundaries	References to the specific geographic boundaries of the research project area(s).
Features	References to natural and manmade features of the research project area(s).
Ecology	References to wildlife, habitat, and natural systems within a research project area .
**Ecosystem Services**	**Ecosystem Services and the benefits humans receive from nature**
ES focus	References to the specific ecosystem services that were the focus of the research.
Who determined	References to who determined which ecosystem services were focused on.
**Problem Definition**	**References to who defined the research. Stating or defining the nature of the questions and who identified them**
Program of origin	References to aspects of the research defined by a specific Research Program.
ORD driven	References to aspects of the research defined by ORD.
Program_Regional driven	References to aspects of the research defined by Regional or Program Offices.
Stakeholder driven	References to aspects of the research defined by stakeholders.

Decision attachment	References to aspects of the research defined by existing decisions.
**Research Mechanism**	**How the research was carried out- agency or means by which the research actions were accomplished**
Partnership	References to partnerships with stakeholders as a means for accomplishing research actions.
Program support	References to program support as a means for accomplishing research actions.
RESES_RARE	References to RESES, RARE or ORD extramural funding as a means for accomplishing research actions.
Cooperative	References to a Cooperative Agreement or a Memorandum of Understanding with university or other types of agreements as a means for accomplishing research.
**Collaborators**	**People, groups, organizations and agencies who work jointly on a project or aspects of a project (double-coded to ROLES when referencing specific roles)**
ORD	References to ORD staff as collaborators.
Regional Office	References to EPA Regional Offices and their role in collaboration.
Program Office	References to EPA Program Offices and their role in collaboration.
National Estuary Program	References to NEPs and their role in collaboration. (create reference list of partnerships for NEPs - PSP, T, SJB, MB)

University partner	References to University departments, staff or students and their role in collaboration.
Community group	References to community groups and their role in collaboration.
Enviro NGO	References to environmental non-governmental organizations and their roles in collaboration.
Local gov	References to local government, including municipal, county or regional government, and their role in collaboration.
State gov	References to state government agencies or organizations and their role in collaboration.
Tribes	References to native tribal authorities and their role in collaboration.
Other	References to other persons or groups and their role in collaboration.
**Input**	**Input from community stakeholders attached to a decision process**
Reality_input collected	References to community input informing the process (actual input).
Planning_input	References to the plan of input collection and/or use in the research-what and how.
Who provided	References to which stakeholders provided input (connected to input or planning for input).
**Stakeholders**	**An individual or group who possesses an interest, obligation, right, or concern in the decisions or outcomes of decisions in relation to the projects**

Who	References to the identity or description of stakeholder groups.
How identified	References to how the project team identified stakeholders.
Invited	References to if stakeholders were invited to be part of the process (yes or no).
How invited	References to how stakeholders were invited to the process. If certain stakeholders were not invited, references to why those stakeholders were not invited.
Communication methods	References to how stakeholder groups were communicated with. Code references to unspecific stakeholder communication here.
One way	Information transfer from sender to receiver (versus two way).
Two way	Transmission of information in which both parties send and receive information (versus one way).
Ongoing	Continuing communication (versus one-off).
One off	One-time communication (versus ongoing).
Other	
**Roles**	**The function assumed or part played by a person or group in a particular situation**
ORD	References to the role of ORD.
Regional Office	References to the role of an EPA Regional Office.

Program Office	References to the role of a Program Office.
National Estuary Program	References to the role of a NEP.
University partner	References to the role of a university partner.
Community group	References to the role of a community group.
Enviro NGO	References to the role of an environmental non-governmental organization.
Local gov	References to the role of local government.
State gov	References to the role of state government and agencies.
Tribes	References to the role of Native American or other indigenous tribes.
Other	References to roles fulfilled by other persons or groups for the/a project.
**Conceptual frameworks_models_EPA tools**	**References to conceptual frameworks and models use (FEGS approach, SDM, etc.)**
Planning_frameworks_models_tools	References in project plans to conceptual frameworks, models, or EPA tools and their anticipated use. Planning for conceptual frameworks, models, and EPA tools - what and how.
Reality_frameworks_models_tools	References to how conceptual frameworks, models, or EPA tools were utilized in a project.
**Conclusions or Outcomes**	**Conclusions of research/project efforts**

Expected findings and applications	References to anticipated outcomes and products. (ex: "used to inform future management decisions").
Findings	References to actual research findings.
Applications	References to specific applications of research findings.
Products_tangible	References to specific tangible products resulting from the research.
**Methods**	**The TANGIBLE scientific methodology employed by EPA scientists in their research.**
Planned_methods _what and how	References in research plans to specific methodology and its anticipated use.
Reality_methods implementation	References to how specific methodology was used in research.
